# Clinical decision support for bipolar depression using large language models

**DOI:** 10.1038/s41386-024-01841-2

**Published:** 2024-03-13

**Authors:** Roy H. Perlis, Joseph F. Goldberg, Michael J. Ostacher, Christopher D. Schneck

**Affiliations:** 1 Center for Quantitative Health and Department of Psychiatry, Massachusetts General Hospital, Boston, MA USA; 2 Department of Psychiatry, Harvard Medical School, Boston, MA USA; 3 Department of Psychiatry, Mt. Sinai School of Medicine, New York, NY USA; 4 Department of Psychiatry and Behavioral Sciences, Stanford University School of Medicine, Palo Alto, CA USA; 5 Department of Psychiatry, University of Colorado School of Medicine, Aurora, CO USA

**Keywords:** Translational research, Predictive markers

## Abstract

Management of depressive episodes in bipolar disorder remains challenging for clinicians despite the availability of treatment guidelines. In other contexts, large language models have yielded promising results for supporting clinical decisionmaking. We developed 50 sets of clinical vignettes reflecting bipolar depression and presented them to experts in bipolar disorder, who were asked to identify 5 optimal next-step pharmacotherapies and 5 poor or contraindicated choices. The same vignettes were then presented to a large language model (GPT4-turbo; gpt-4-1106-preview), with or without augmentation by prompting with recent bipolar treatment guidelines, and asked to identify the optimal next-step pharmacotherapy. Overlap between model output and gold standard was estimated. The augmented model prioritized the expert-designated optimal choice for 508/1000 vignettes (50.8%, 95% CI 47.7–53.9%; Cohen’s kappa = 0.31, 95% CI 0.28–0.35). For 120 vignettes (12.0%), at least one model choice was among the poor or contraindicated treatments. Results were not meaningfully different when gender or race of the vignette was permuted to examine risk for bias. By comparison, an un-augmented model identified the optimal treatment for 234 (23.0%, 95% CI 20.8–26.0%; McNemar’s *p* < 0.001 versus augmented model) of the vignettes. A sample of community clinicians scoring the same vignettes identified the optimal choice for 23.1% (95% CI 15.7–30.5%) of vignettes, on average; McNemar’s *p* < 0.001 versus augmented model. Large language models prompted with evidence-based guidelines represent a promising, scalable strategy for clinical decision support. In addition to prospective studies of efficacy, strategies to avoid clinician overreliance on such models, and address the possibility of bias, will be needed.

## Introduction

Depressive episodes during bipolar disorder contribute substantially to both morbidity and mortality risk [1]. Yet despite a broadening range of evidence-based interventions for this phase of illness, appropriate treatment of bipolar depression remains challenging and controversial [2] even for physicians focused on specialty care. For example, an International Society for Bipolar Disorder workgroup acknowledged multiple areas of disagreement [3], and efforts to examine consensus among clinicians have identified persistent heterogeneity in treatment approaches [4, 5].

In an effort to create a standard of care, treatment guidelines based on systematic reviews of evidence have proliferated [6–10], albeit with the notable absence of the American Psychiatric Association. Although such guidelines provide an outline of reasonable strategies, their application for personalizing treatment based on an individual patient’s prior treatment and illness history, as well as their preferences, remains difficult.

Large language models have demonstrated proficiency across medicine [11], from responding to clinical examination questions to identifying rare or difficult-to-solve clinical scenarios [12]. In a preliminary study, we demonstrated that without fine-tuning or other further training, one model could approximate the performance of clinicians in recommending next-step treatments in major depression – but also that the model made potentially harmful errors [13].

In the present study, we describe an approach to clinical decision support that augments a standard large language model with a prompt incorporating a summary of evidence-based guidelines to elicit a set of next-step pharmacologic options. This approach is similar to retrieval-augmented generation models that attempt to match a query with fragments of text from a set of documents, but takes advantage of the capacity of newer large language models to include a large amount of text in the model prompt itself, obviating the need to parse a document. Our primary objective was to compare this strategy, which allows flexibility in incorporating evidence-based recommendations in clinical practice versus purely algorithmic prescribing, to expert consensus recommendations. For comparison, we also examined the extent to which an un-augmented large language model (i.e., without additional knowledge), could approximate expert consensus recommendations. We also examined performance of a group of community prescribers, as an approximation of community standard of care. To address the possibility of bias, we further considered the extent to which model outputs may be biased by gender and race.

## Materials and methods

### Vignette generation

We applied a probabilistic model, adapted from approximate prevalences of treatment in the electronic health records of 2 academic medical centers and affiliated community hospitals, to generate 50 vignettes for individuals with bipolar 1 or 2 disorder experiencing a current major depressive episode. Each vignette included age and gender (the latter randomly assigned with 50% probability between men and women), with race randomly assigned with 50% probability between individuals who were white and Black. Sociodemographic assignments were intended to make vignettes more realistic while maintaining large enough subgroups to allow secondary analysis to examine bias; as such, we elected not to include other gender, race, or ethnicity categories. Vignettes also included medical and psychiatric comorbidities, current and past medications, and features of past illness course. (See Supplementary Materials for vignette template and example vignette).

### Vignette evaluation

Optimal treatment options for each vignette were collected from 3 clinicians with bipolar disorder expertise, each with more than 20 years of mood disorder practice and experience leading mood disorder clinics. A community clinician comparator group was collected via internet survey of clinicians who treat individuals with bipolar disorder who were participating in a continuing medical education program, invited to participate by email. The clinicians were offered entry into a lottery as incentive to participate. All surveys were administered via Qualtrics.

The expert clinicians were presented with all 50 vignettes in random order, with gender and race randomly permuted. For each vignette, they were asked to identify and rank the 5 best next-step treatments to consider, and the 5 worst or contraindicated next-step treatments. Expert-defined optimal treatment for a given vignette was assigned on the basis of mean ranking of each medication. Poor treatment was assigned on the basis of appearing in a list of poor options from at least one expert. The surveyed clinicians were similarly presented with 20 randomly-selected vignettes in random order drawn from the 50, with gender and race randomly permuted.

All respondents signed written informed consent prior to completing the survey, which was approved by the Mass General-Brigham Institutional Review Board.

### Model design

The augmented model used GPT-4 with a prompt incorporating 3 sections (Supplementary Materials). The first presented the context and task; the second summarized the knowledge to be used in selecting treatment; and the third presented the clinical vignette. The knowledge incorporated an excerpt from the US Veterans Administration 2023 guidelines for bipolar disorder [6] relating to pharmacologic management of depression. For primary result generation, the model prompt asked to return a ranked list of the best 5 next-step interventions. For exploration of these results, an alternate prompt (Supplementary Materials) asked that each intervention be justified by citing the rationale relied upon for recommendation.

As a comparator (the ‘base model’), we repeated scoring using a shortened version of the augmented model prompt to elicit model recommendations without relying on additional knowledge (Supplementary Materials).

All models used GPT-4 turbo (gpt-4-1106-preview), with temperature set at 0 to generate the most deterministic (i.e., least random) results, and context reset prior to each vignette. (While temperature can be applied to increase apparent creativity in generative models, we presumed that creativity in treatment selection would decrease replicability and transparency). Each vignette was presented a total of 20 times, with gender and race permuted (that is, each combination of male or female, and Black or white, was presented 5 times). Web queries and code execution by GPT-4, as well as use of additional agents, were inactivated to ensure that all responses used only the knowledge provided plus the prior training.

### Analysis

In the primary analysis, we compared the augmented model to the expert selections, evaluating how often this model identified the expert-defined optimal medication choice. To examine whether this augmentation was useful, we then compared an unaugmented model (i.e., the LLM without inclusion of guideline knowledge) to expert selections, again evaluating how often it selected the optimal choice. Performance of the two models was compared using McNemar’s test for agreement. To facilitate comparison to community practice, we also calculated the proportion of vignettes for which community clinician choices matched expert selections. For the augmented and base model, as well as the community clinicians, we also evaluated how often models or clinicians selected at least one expert-defined poor medication choice.

To evaluate the possibility of biased responses, we also examined model responses stratified by 4 gender-race pairs (Black men, Black women, white men, white women). To compare the 4 groups, we used Cochrane’s Q test (a generalized form of McNemar’s test), followed by post-hoc pairwise tests using McNemar’s test with Bonferroni-corrected *p*-values. This approach is analogous to using ANOVA with post hoc pairwise tests.

We conducted two post hoc sensitivity analyses. First, as one of the experts had contributed to guideline development, we examined the effect of excluding that expert, to ensure the overlap (between guideline writing and expert opinion) did not inflate concordance between augmented model output and expert results. Second, we examined the performance of psychiatrist prescribers alone, rather than including all clinicians.

In reporting model performance, we elected to report proportion of times that the optimal expert choice was selected as the first choice, both as proportion and as Cohen’s kappa. We adopted this strategy to maximize transparency and interpretability, and because comparisons of ranked lists, when the rankings are partial (i.e., not all options are scored), is not a well-defined area of statistical methodology [14, 15]. Secondarily, we reported how often the experts’ top choice was among the top 3, or top 5, provided by the model. We further examined degree of overlap between the 5 model choices and the 5 expert choices. Finally, we compared the models’ top 5 choices to the list of poor choices identified by the experts.

## Results

Agreement between the 3 expert clinicians measured by Cohen’s kappa ranged from 0.10 (95% CI 0.01–0.18) to 0.22 (95% CI 0.11–0.33). For the augmented model, Cohen’s kappa with the expert consensus was 0.31 (95% CI 0.28–0.35). For 508 vignettes (50.8%), the model identified the optimal treatment; for 844 (84.4%) the optimal treatment was among the top 3 nominated by the model; and for 949 (94.9%) the optimal treatment was among the top 5. Mean overlap between the model’s selections and the expert selections was 3.7 (SD 0.6) – that is, on average, 3.7 of the model’s medications appeared among the expert top 5. Conversely, for 120 (12.0%) of vignettes, the model selected a medication considered by the experts to be a poor choice, or contraindicated.

In subgroup analyses of the 4 gender-race pairs, model performance exhibited differences of modest magnitude (Table 1; Supplementary Table 1) that were statistically significant (*p* = 0.02). In post-hoc pairwise contrasts using McNemar’s test, model performance in Black women was significantly poorer than white men (Bonferroni-corrected *p* = 0.03).

**Table 1:** Comparison of ratings from models or clinicians to expert recommendation.

Source	Top choice						Poor choice (a)			
	Kappa	95% CI	*n*	%	[95%	CI]	*n*	%	[95%	CI]
Augmented Model	0.31	0.28–0.35	508	50.8%	47.7%	53.9%	120	12.0%	10.0%	14.0%
Black Man	0.31	0.24–0.38	126	50.4%	44.2%	56.6%	29	11.6%	7.6%	15.6%
Black Woman	0.28	0.21–0.35	121	48.4%	42.2%	54.6%	26	10.4%	6.4%	14.4%
White Man	0.34	0.27–0.41	133	53.2%	47.0%	59.4%	33	13.2%	9.4%	17.0%
White Woman	0.32	0.25–0.39	128	51.2%	45.0%	57.4%	32	12.8%	8.6%	17.0%
Base Model	0.09	0.07–0.12	234	23.4%	20.8%	26.0%	108	10.8%	8.9%	12.7%
Black Man	0.06	0.01–0.11	51	20.4%	15.2%	25.6%	25	10.0%	6.2%	13.8%
Black Woman	0.11	0.06–0.16	61	24.4%	19.4%	29.4%	26	10.4%	6.7%	14.1%
White Man	0.11	0.05–0.16	63	25.2%	19.9%	30.5%	31	12.4%	8.6%	16.2%
White Woman	0.09	0.04–0.14	59	23.6%	18.2%	29.0%	26	10.4%	6.3%	14.5%
All Clinicians (a)	0.09	0.03–0.15		23.1%	15.7%	30.5%		24.4%	17.7%	31.1%
Psychiatrists (b)	0.11	0.00–0.22		23.4%	11.9%	34.9%		23.9%	12.3%	35.6%

(a) among ‘poor choice’ vignettes for augmented model, *n* = 0 vignettes with more than 1 poor choice; among base model, *n* = 32 vignettes in which 2 of 5 top-ranked choices were poor (7, 9, 8, and 8, respectively).

(b) values calculated as mean of individual clinician results.

**Fig. 1:**
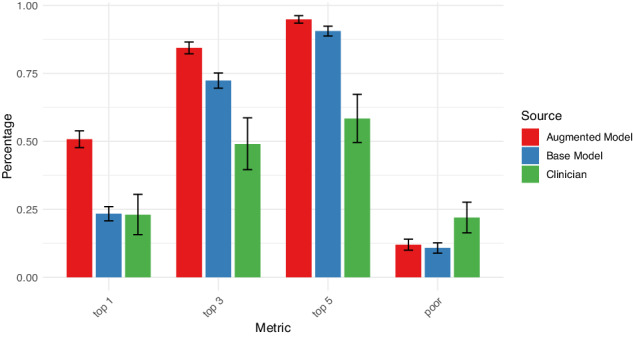
Comparison of augmented language model, base model, and clinician medication selections. Bars indicate proportion selecting expert-defined optimal or poor treatment, with 95% confidence interval.

For comparison, we repeated these analysis with an unaugmented model, using a prompt that excluded any specific knowledge of guidelines (Table 1, bottom). Cohen’s kappa with the expert consensus was 0.09 (95% CI 0.07–0.12). For 234 (23.4%) of vignettes, the model identified the optimal treatment, a result significantly poorer than for the augmented model (McNemar’s *p* < 0.001); for 724 (72.4%) the optimal treatment was among the top 3 nominated by the model; and for 906 (90.6%) the optimal treatment was among the top 5. Mean overlap between the model’s selections and the expert selections was 2.8 (SD 0.7). For 108 (10.8%) of vignettes, the model selected a medication considered by the experts to be a poor choice, or contraindicated; this result was not significantly different from the augmented model (McNemar’s *p* = 0.4). Figure 1 summarizes these contrasts. Results were similar when one of the 3 experts who had contributed to guideline development was excluded from analysis (Supplementary Table 2). In subgroup analyses constraining vignettes to a single gender, or either white or Black race, no significant differences in model performance were identified (*p* = 0.17).

We next compared community clinicians with experience in treating bipolar disorder to the expert consensus. A total of 27 prescribing clinicians participated (10 psychiatrists (37%), 12 psychiatric nurse practitioners (44%), 2 non-psychiatric (7%), 2 physician assistants (7%), and 1 primary care physician (4%). They reported a mean of 12.7 (SD 10.3) years in practice, and treating a mean of 9.2 (SD 5.1) individuals with bipolar disorder per week. Each vignette was scored by a median of 4 [25–75% interval 3–5] clinicians. Table 1, Fig. 1, and Supplementary Table 1 summarize community clinician performance. Mean kappa was 0.07 (95% CI −0.15 to 0.29); a mean of 23.0% ranked the optimal medication first, 49.0% ranked the optimal medication among their top 3, and 58.4% among their top 5 choices. Mean overlap between clinician choices and expert selections was 2.2 (SD 1). A mean of 22.0% selected at least one poor or contraindicated medication among their top 5 choices. Secondary analysis including only vignettes scored by the 10 psychiatrists yielded similar results (Table 1, bottom).

Finally, to demonstrate how such a model could be applied in clinical settings, we modified the prompt to return an explanation for each treatment selection. (Supplementary Materials) This chat-based approach also illustrates a more interactive application, including consideration in real time of clinician and patient preference.

## Discussion

In this comparison of a decision support tool integrating treatment guidelines with a large language model via retrieval-augmented generation, we found that a model augmented with an excerpt from published treatment guidelines selected the next-step medication considered optimal by clinical experts based on a curated set of clinical vignettes, more than half the time. This performance compared favorably with a base or un-augmented model (i.e., relying on GPT-4 with no additional knowledge), and with a sample of community clinicians experienced in treatment of bipolar disorder, although agreement between the augmented model and expert ratings was only fair. The discordance between community clinician performance and expert choices likely reflects the continuing difficulty in treatment decision-making for this aspect of bipolar disorder [2], with heterogeneity in expert opinion [3] as well as clinical practice [4, 5]. Indeed, the modest agreement among experts underscores the challenge in developing clinical decision support in areas where the gold standard may be difficult to define.

We also investigated bias by comparing model performance in vignettes subsetted by gender or race. While differences were modest in numeric terms, there was some evidence of bias in results: an omnibus test for differences between gender and race group suggested significant differences in performance in terms of differences in ability to select optimal treatment. An advantage of inclusion of knowledge for the model to operate on, akin to retrieval augmented generation on a smaller scale, is that it enables greater visibility into reference materials (i.e., knowledge to be incorporated in decisionmaking) than the baseline models. There is no obvious reason that the guideline text would introduce bias, such that this may represent type 1 error. Nonetheless, it underscores the importance of careful characterization of models in terms of fairness both before and after deployment. An evolving literature suggests that when LLM’s are asked to perform clinical tasks, they may exhibit subtle biases – for example, in associating race with diagnoses when asked to generate clinical vignettes [16]. On the other hand, in other contexts LLM’s may exhibit less biased responses [17].

Our results are difficult to compare to prior investigations in psychiatry. In one prior report [13], we described the use of GPT4 for antidepressant selection based on a small set of previously-validated vignettes, without any augmentation. That study, which did not employ any additional knowledge in prompting, was notable for the high rates at which the model selected poor or contraindicated options – in particular, at least one such choice was included among optimal treatments in 48% of vignettes.

The observation that incorporating knowledge directly in prompting diminishes likelihood of contraindicated recommendations is significant in light of prior work indicating that psychiatric clinicians are susceptible to being influenced to make incorrect choices by artificial intelligence tools [18]. It is thus critical to consider, not only how often models succeed, but also their mode and frequency of failure. While our results suggest the utility of augmenting model knowledge in diminishing this risk, we note that the model did still yield some responses which, if adopted, could prove to be harmful to patients.

The use of a retrieval-augmented generation architecture for large language models is a rapidly-evolving strategy for more transparently incorporating knowledge in such models (for an example in a clinical context, see Zakka [19]). For this proof-of-concept study, we incorporated a guideline document directly in the prompt itself, rather than requiring retrieval, a strategy that is increasingly feasible as the context window for large language models grows. This highly-extensible architecture can readily incorporate additional perspectives to more closely approximate clinical practice. For example, documents could encompass clinician preferences or standards of care in a particular health system; others could encompass individual patient preferences, such as adverse effects felt to be more or less acceptable. Our work seeks to provide a baseline, and a set of curated vignettes, that can be applied for such studies. A key question for future investigations will be the extent to which the incorporation of guidelines improves treatment selections in other clinical contexts.

### Limitations

This study has multiple limitations. While the vignettes are derived from real-world cases and intended to reflect clinical descriptions, we cannot exclude the possibility that critical information omitted from the vignettes would have improved or degraded prediction. Further work will be required to better understand the sensitivity of prediction to extent of clinical detail. We did include potential distractors (e.g., occupation, living situation) and medical and psychiatric comorbidities to enhance the realism of the vignettes. In addition, while the gold standard reflects annotation by expert clinicians, prospective investigation will be required to understand whether approximating expert opinion is the optimal way to improve outcomes – i.e., to establish the efficacy and safety of LLM-based decision support tools. While the US FDA has addressed international standards for software as a medical device [20, 21], the applicability of these standards in this context remains to be established. Finally, additional clinician data will be valuable in benchmarking model performance against more generalizable cohorts; in light of the small number of clinicians who participated, we cannot exclude the possibility that other groups would exhibit substantially greater, or poorer, performance.

## Conclusion

With these caveats in mind, our results nonetheless demonstrate the potential utility of a straightforward, interpretable approach to integrating treatment guidelines with clinical context to yield a decision-support tool. In light of the known challenges of treating bipolar depression, as the augmented model performed better on average than a sample of community clinicians, randomized trials to determine whether our augmented model can improve clinical outcomes, without increasing risk, merit consideration. More broadly, our results suggest the potential utility of applying large language models to provide a guideline-based standard of care in clinical settings, allowing transparency and portability in development of these decision support tools.

### Supplementary information

Supplemental Material

## Data Availability

All vignettes, templates, and surveys used for this study are available from the corresponding author for non-commercial use.
