# Supplementary material for: Clinical decision support for bipolar depression using large language models
Source: Neuropsychopharmacology. 2024 Mar 13;49(9):1412–6. doi: 10.1038/s41386-024-01841-2 (PMC11251032; doi:10.1038/s41386-024-01841-2)
Supplement: Supplementary file 1 — Supplemental Material [file 41386_2024_1841_MOESM1_ESM.pdf]

Supplemental Materials
Page 1-4: Supplemental Text
Page 5: Supplemental Table 1: Comparison of ratings from models or clinicians to expert annotation
Page 6: Supplemental Table 2: Comparison of ratings from models or clinicians to expert annotation, excluding one expert

A. Supplemental Text

1. Vignette template:
This is a xx year old {Gender} who is {Race}, with a history of bipolar {1/2} disorder and {medical comorbidity}, {psychiatric comorbidity}, {xx} prior hospitalizations, and {xx} prior suicide attempts. The patient presents with {duration} of {course} depressive symptoms, including {prominent symptoms}. Current psychiatric medications include {current psychiatric medications}, {current comorbidity medications}. Past medications include {past psychiatric medications, with reason for discontinuation for each}. {Relevant prior course}. The patient {occupation/employment} and lives with {home situation}.

2. Vignette example:
This is a 37 year old man who is white, with a history of bipolar 1 disorder and type 2 diabetes mellitus, as well as a past history of alcohol use disorder, panic attacks, four prior hospitalizations, and two prior suicide attempts. The patient presents with nine months of persistent depressive symptoms, including fatigue. Current psychiatric medications include lithium (1200mg), lurasidone (40mg), venlafaxine (75mg), and clonazepam (1mg). Additional medications include metformin. Past medications include quetiapine, which was discontinued due to weight gain, and ziprasidone, which was discontinued due to restlessness. The prior course is otherwise notable for psychotic symptoms during past depressions. The patient is a graduate student and lives with a partner.

3. Prompt template:
"You are an expert psychiatrist teaching medical students. For the following vignette, return only a rank-ordered list of the 5 best medicines to consider adding or substituting as next steps, with #1 being the best. Do not include optimizing doses or anything except medication names. Your response should have the form: medicine1,medicine2,medicine3,medicine4,medicine5. This is very important for me, please take your time and go step by step. Do not include any additional text. {evidence_prompt}... The vignette is:" {vignette}

4. Illustration of use of augmented model in interactive context:

Prompt: *You are an expert psychiatrist teaching medical students. For the following vignette, return a rank-ordered list of the 5 best medicines to consider adding or*

substituting as next steps, with #1 being the best. Do not include optimizing doses or anything except medication names. Your response should have the form: medicine1,medicine2,medicine3,medicine4,medicine5. This is very important for me, please take your time and go step by step. Then, explain your rationale for each choice, referring to the specific relevant text from the knowledge provided. Use this knowledge to decide, before relying on anything else...

Based on the provided knowledge and the patient's clinical history, the rank-ordered list of the 5 best medicines to consider adding or substituting as next steps would be:

1. Lamotrigine
2. Cariprazine
3. Lumateperone
4. Lurasidone
5. Olanzapine

Rationale for Each Choice:

1. **Lamotrigine**: The guidelines suggest lamotrigine in combination with lithium or quetiapine for acute bipolar depression (guideline 15). Although there is insufficient evidence to recommend lamotrigine as monotherapy (guideline 14), its combination with lithium, which the patient is already taking, could be beneficial, especially considering its tolerability and the patient's history of substance use disorder.
2. **Cariprazine**: This is suggested as an alternative to quetiapine based on patient preference and characteristics for acute bipolar depression (guideline 13). Since quetiapine was discontinued due to sedation in this patient, cariprazine could be a suitable alternative, particularly considering its efficacy in bipolar depression.
3. **Lumateperone**: Also suggested as an alternative to quetiapine for monotherapy in acute bipolar depression (guideline 13). Lumateperone may be beneficial given its newer antipsychotic profile and potentially favorable side effect profile, which could be important given the patient's past side effects with quetiapine.
4. **Lurasidone**: Another alternative to quetiapine for monotherapy in acute bipolar depression (guideline 13). Lurasidone's effectiveness in bipolar depression and relatively favorable metabolic profile make it a good option, considering the patient's past medication experiences.
5. **Olanzapine**: While effective for bipolar depression, olanzapine is ranked lower due to its side effect profile (guideline 13). It's an option if other medications are not effective or suitable, but its potential for weight gain and metabolic issues

should be considered, especially in a patient with a past history of substance use disorder.

In this case, the prioritization of lamotrigine aligns with the guidelines and the patient's specific clinical needs, including a history of bipolar disorder, past substance use disorder, and prior medication side effects. The choices also reflect the need to balance efficacy in treating bipolar depression with the potential for side effects.

Prompt: *What about using fluoxetine?*

Using fluoxetine in the context of this patient with bipolar II disorder should be approached with caution. According to the guidelines provided, there is insufficient evidence to recommend for or against antidepressants to augment treatment with second-generation antipsychotics or mood stabilizers for acute bipolar depression (guideline 17). Additionally, there is insufficient evidence to recommend for or against antidepressants or lamotrigine as monotherapy for acute bipolar depression (guideline 14).

In the specific case of this patient, several factors need to be considered:

1. **Risk of Mania**: Antidepressants, including fluoxetine, can potentially trigger manic or hypomanic episodes in patients with bipolar disorder. Given the patient's history of bipolar II disorder and past psychotic symptoms, this risk should be carefully weighed.
2. **Current Medication Regimen**: The patient is already on sertraline, another antidepressant. Adding fluoxetine or substituting sertraline with fluoxetine might not significantly change the treatment strategy or outcome, especially if sertraline is well-tolerated.
3. **Individual Response and Side Effects**: Every patient's response to medication can be different. If the patient has not responded well to sertraline or if there are specific side effects, fluoxetine might be considered. However, this should be based on a careful assessment of the patient's history and current clinical status.
4. **Monitoring and Management**: If fluoxetine or any antidepressant is used in a patient with bipolar disorder, it should be done under close monitoring for any signs of mood destabilization, particularly manic or hypomanic symptoms.

In summary, while fluoxetine could be considered, its use in bipolar depression, particularly bipolar II, requires careful consideration and close monitoring, aligning with the guidelines and the patient's specific clinical profile.

Prompt: *What about restarting quetiapine?*

Restarting quetiapine for this patient, who has a history of bipolar II disorder and is currently experiencing depressive symptoms, aligns with the guidelines you provided:

1. **Quetiapine for Bipolar Depression**: Guideline 12 strongly recommends quetiapine as monotherapy for acute bipolar depression. This recommendation makes quetiapine a primary consideration for patients experiencing bipolar depression.
2. **Patient's Past Experience with Quetiapine**: However, it's important to note that the patient discontinued quetiapine previously due to sedation. This side effect is a common reason for discontinuation and should be carefully reconsidered. If the sedative effects of quetiapine were significantly impairing the patient's quality of life or daily functioning, this could be a limiting factor for its re-introduction.
3. **Balancing Efficacy and Tolerability**: The decision to restart quetiapine should involve a balance between its efficacy for bipolar depression and the patient's ability to tolerate its side effects. If the sedation was manageable or could be mitigated (e.g., by adjusting the timing of the dose), it might be reasonable to retry quetiapine, especially given its strong recommendation in the guidelines.
4. **Considering Other Medications**: The patient is currently on lithium, brexpiprazole, and sertraline. If quetiapine is reintroduced, it would be in combination with these medications. The interaction and cumulative effect of these medications, especially in the context of bipolar depression, should be carefully assessed.
5. **Monitoring and Adjustment**: If quetiapine is restarted, careful monitoring for sedation and other side effects, as well as its effectiveness in managing depressive symptoms, is essential. Adjustments in dosing or timing, or even considering other medications (like lamotrigine, cariprazine, lumateperone, lurasidone, or olanzapine as per your guidelines) might be necessary based on the patient's response.

In conclusion, while restarting quetiapine is a viable option and aligns with the guidelines for treating acute bipolar depression, the decision should be made considering the patient's past experiences with the medication, current medication regimen, and the need for careful monitoring and potential dose adjustments.

Table 1. Comparison of ratings from models or clinicians to expert annotation

	Top choice						Among top 3				Among top 5				Overlap of top 5		Poor choice (a)			
**Source**	**Kappa**	**95% CI**	**n**	**%**	**[95%**	**CI]**	**n**	**%**	**[95%**	**CI]**	**n**	**%**	**[95%**	**CI]**	**Overlap**	**SD**	**n**	**%**	**[95%**	**CI]**
Augmented Model	0.31	0.28-0.35	508	50.8%	47.7%	53.9%	844	84.4%	82.2%	86.6%	949	94.9%	93.5%	96.3%	3.7	0.6	120	12.0%	10.0%	14.0%
Black Man	0.31	0.24-0.38	126	50.4%	44.2%	56.6%	210	84.0%	79.5%	88.5%	237	94.8%	92.1%	97.5%	3.7	0.6	29	11.6%	7.6%	15.6%
Black Woman	0.28	0.21-0.35	121	48.4%	42.2%	54.6%	210	84.0%	79.5%	88.5%	236	94.4%	91.6%	97.2%	3.7	0.6	26	10.4%	6.4%	14.4%
White Man	0.34	0.27-0.41	133	53.2%	47.0%	59.4%	211	84.4%	79.9%	88.9%	237	94.8%	91.9%	97.7%	3.7	0.6	33	13.2%	9.4%	17.0%
White Woman	0.32	0.25-0.39	128	51.2%	45.0%	57.4%	213	85.2%	80.7%	89.7%	239	95.6%	92.8%	98.4%	3.7	0.6	32	12.8%	8.6%	17.0%
Base Model	0.09	0.07-0.12	234	23.4%	20.8%	26.0%	724	72.4%	69.6%	75.2%	906	90.6%	88.8%	92.4%	2.8	0.7	108	10.8%	8.9%	12.7%
Black Man	0.06	0.01-0.11	51	20.4%	15.2%	25.6%	176	70.4%	64.9%	75.9%	225	90.0%	86.4%	93.6%	2.8	0.7	25	10.0%	6.2%	13.8%
Black Woman	0.11	0.06-0.16	61	24.4%	19.4%	29.4%	183	73.2%	67.5%	78.9%	226	90.4%	86.7%	94.1%	2.8	0.7	26	10.4%	6.7%	14.1%
White Man	0.11	0.05-0.16	63	25.2%	19.9%	30.5%	179	71.6%	66.1%	77.1%	226	90.4%	86.7%	94.1%	2.8	0.7	31	12.4%	8.6%	16.2%
White Woman	0.09	0.04-0.14	59	23.6%	18.2%	29.0%	186	74.4%	68.8%	80.0%	229	91.6%	87.9%	95.3%	2.8	0.7	26	10.4%	6.3%	14.5%
All Clinicians (b)	0.09	0.03-0.15		23.1%	15.7%	30.5%		47.6%	38.3%	56.9%		58.4%	50.1%	66.8%	2.2	1		24.4%	17.7%	31.1%
Psychiatrists only (b)	0.11	0.00-0.22		23.4%	11.9%	34.9%		55.7%	42.2%	69.2%		65.3%	52.1%	78.4%	2.4	1		23.9%	12.3%	35.6%

(a) among 'poor choice' vignettes for augmented model, n=0 vignettes with more than 1 poor choice; among base model, n=32 vignettes in which 2 of 5 top-ranked choices were poor (7, 9, 8, and 8, respectively)

(b) values calculated as mean of individual clinician results

Supplemental Table. Comparison of ratings from models or clinicians to expert recommendation, limited to 2 experts

	Top choice						Among top 3				Among top 5				Overlap of top 5		Poor choice (a)			
**Source**	**Kappa**	**95% CI**	**n**	**%**	**[95%**	**CI]**	**n**	**%**	**[95%**	**CI]**	**n**	**%**	**[95%**	**CI]**	**Overlap**	**SD**	**n**	**%**	**[95%**	**CI]**
Augmented Model	0.42	0.39-0.45	592	59.2%	56.2%	62.2%	852	85.2%	83.0%	87.4%	917	91.7%	90.0%	93.4%	3.6	0.6	111	11.1%	9.2%	13.0%
Black Man	0.43	0.37-0.49	150	60.0%	53.9%	66.1%	214	85.6%	81.2%	90.0%	230	92.0%	88.6%	95.4%	3.6	0.6	26	10.4%	6.5%	14.3%
Black Woman	0.41	0.35-0.47	146	58.4%	52.3%	64.5%	211	84.4%	80.0%	88.8%	229	91.6%	88.2%	95.0%	3.6	0.7	31	12.4%	8.6%	16.2%
White Man	0.42	0.36-0.49	149	59.6%	53.5%	65.7%	214	85.6%	81.1%	90.1%	229	91.6%	88.2%	95.0%	3.6	0.6	28	11.2%	7.1%	15.3%
White Woman	0.42	0.35-0.48	147	58.8%	52.7%	64.9%	213	85.2%	80.8%	89.6%	229	91.6%	88.2%	95.0%	3.6	0.6	26	10.4%	6.5%	14.3%
Base Model	0.23	0.20-0.25	358	35.8%	32.8%	38.8%	651	65.1%	62.1%	68.1%	871	87.1%	85.0%	89.2%	2.7	0.7	122	12.2%	10.2%	14.2%
Black Man	0.23	0.17-0.28	87	34.8%	28.9%	40.7%	157	62.8%	56.9%	68.7%	215	86.0%	81.8%	90.2%	2.8	0.6	28	11.2%	7.1%	15.3%
Black Woman	0.20	0.15-0.25	82	32.8%	26.9%	38.7%	160	64.0%	58.0%	70.0%	218	87.2%	82.9%	91.5%	2.7	0.7	35	14.0%	10.1%	17.9%
White Man	0.24	0.18-0.30	95	38.0%	32.2%	43.8%	166	66.4%	60.4%	72.4%	218	87.2%	83.1%	91.3%	2.7	0.7	29	11.6%	7.3%	15.9%
White Woman	0.23	0.18-0.29	94	37.6%	31.6%	43.6%	168	67.2%	61.3%	73.1%	220	88.0%	83.9%	92.1%	2.7	0.7	30	12.0%	8.0%	16.0%
Clinician (b)	14.3	0.08-0.20		28.0%	20.7%	35.3%		48.2%	39.0%	57.5%		56.2%	48.2%	64.3%	2.2	1		22.0%	15.9%	28.1%

(a) among 'poor choice' vignettes for augmented model, n=0 vignettes with more than 1 poor choice; among base model, n=45 vignettes in which 2 of 5 top-ranked choices were poor (12,10,11, and 12, respectively)
(b) values calculated as mean of individual clinician results
